# Anhedonic Type Behavior and Anxiety Profile of Wistar-UIS Rats Subjected to Chronic Social Isolation

**DOI:** 10.3389/fnbeh.2021.663761

**Published:** 2021-05-28

**Authors:** María Camila Acero-Castillo, María Camila Ardila-Figueroa, Silvia Botelho de Oliveira

**Affiliations:** ^1^Psychology, Universidad Pontificia Bolivariana Sectional Bucaramanga, Santander, Colombia; ^2^Neurosciences and Behavior, Universidad Pontificia Bolivariana Sectional Bucaramanga, Santander, Colombia; ^3^Health Sciences, Universidade de Brasilia, Brasilia, Brazil; ^4^Psychology, Universidade Estadual Paulista, São Paulo, Brazil; ^5^Psychobiology, Universidade de São Paulo, São Paulo, Brazil; ^6^Faculty of Psychology, Universidad Pontificia Bolivariana Sectional Bucaramanga, Santander, Colombia; ^7^Laboratory of Neurosciences and Behavior, Universidad Pontificia Bolivariana Sectional Bucaramanga, Santander, Colombia

**Keywords:** anhedonia, anxiety, depression, chronic social isolation, resocialization

## Abstract

Chronic Social Isolation (CSI) is a model of prolonged stress employed in a variety of studies to induce depression and anxious behavior in rats. The present study aims to evaluate the effect of CSI on male Wistar rats in terms of “anhedonic-type” behavior in the Sucrose Preference Test (SPT) and anxiogenic profile in the elevated-plus-maze (EPM) test, as well as evaluating the effect of resocialization upon sucrose consumption. A total of 24 adolescent male Wistar rats were evaluated. The animals were housed either together (communally) or socially isolated for 21 days, and then exposed for four consecutive days to the SPT test [water vs. a 32% sucrose solution (SS)]. Four days later, they were again subjected to the SPT test (32% vs. 0.7% SS), and then tested on the EPM apparatus 3 days later. Following the completion of the anxiogenic profile of the model, the animals were resocialized for 72 h and then re-tested once again using the SPT (32% vs. 0.7% SS). Twenty-four hours after this final consumption, the animals were euthanized to record the weight of their adrenal glands (AG). It was found that exposure to CSI produces anhedonic-type behavior and an anxiogenic profile in adolescent male rats, as evidenced in both the SPT and EPM tests, as well as in the animals’ physiological stress response. It was also demonstrated that resocialization does not reverse the anhedonic-type behavior, nor the physiological response to stress.

## Introduction

Exposure to stress, especially at early ages, has been shown to be a determining factor in the appearance of depression and anxiety in humans (Starr et al., 2016; Bavley et al., 2017; Gawali et al., 2017; Sangenstedt et al., 2017; Smith and Pollak, 2020). Chronic Social Isolation (CSI) in rodents and non-human primates has been used to model neurobiological alterations linked to such behaviors as depression, anhedonia, and, anxiety (Botelho et al., 2007; Fone and Porkess, 2008; Starr et al., 2016; Shetty and Sadananda, 2017; Mumtaz et al., 2018; Brenes et al., 2020); as well as the development of post-traumatic stress (Berardi et al., 2014). In particular, the Sucrose Preference Test (SPT) has been employed to understand the mechanisms underlying anhedonia. This animal experimentation model is based on the appetitive nature of sweet solutions (Cantora and López Ramírez, 2005; Díaz et al., 2010; Alvarez, 2015), wherein the preference pattern of sucrose solutions (SS) at varying concentrations (0.7%, 1%, 2%, 4%, 5%, 8%, 12%, 16%, 32%, and 35%) is used as an indicator of anhedonic-type behavior (Muscat et al., 1991; Grippo et al., 2003; Cortés et al., 2005; Martínez et al., 2008; Rodríguez et al., 2012; Páez-Ardila and Botelho, 2014; He et al., 2020; Wang et al., 2020; Wright et al., 2020).

In this context, an array of studies have found that animals subjected to CSI consume a greater quantity of high-concentration SS (24%, 32%, and 34%; Muscat et al., 1991; Hall et al., 1997; Brenes Sáenz et al., 2006; Brenes and Fornaguera, 2008, 2009; Wright et al., 2020), even when given the option to choose solutions of various concentrations (Sammut et al., 2002; Martínez et al., 2008). Such alternations in reward–sensitivity have been widely found to be related to the mesolimbic dopaminergic system (Fone and Porkess, 2008; Rodrigues et al., 2011; Noschang et al., 2021). It has also been found that this pattern of consumption can be reversed using antidepressant drugs such as fluoxetine (Brenes and Fornaguera, 2009). The majority of studies have interpreted increased consumption of high-concentration SS as enhanced incentive motivation and reward–sensitivity. In addition, when other models of chronic stress are used, reduced consumption of low–concentration SS has been reported (0.1%, 1%, and 2%; Willner et al., 1987; Papp et al., 2016; Qin et al., 2017; He et al., 2020; Wang et al., 2020). In this context, anhedonia can be defined as a low reward–sensitivity (Rygula et al., 2005; Willner, 2005).

Social isolation also produces anxiety-type behaviors in rodents, which are demonstrated by their performance in tests such as the Open Field and Elevated-Plus-Maze (EPM; Botelho et al., 2007; Pritchard et al., 2013; Kumari et al., 2016; Shetty and Sadananda, 2017; Sequeira-Cordero et al., 2019). The EPM is one of the most widely used models for the evaluation of anxiety-related behavior (Botelho et al., 2007; Amancio-Belmont et al., 2020). It is based upon the natural preference of these animals for dark and protected places, and their anxiety is thus measured by the proportion of test time that they remain in the closed arms of the maze, without coming into contact with the open areas (Pellow et al., 1985; Botelho et al., 2007; Casarrubea et al., 2016). Research indicates that exposure to social isolation in adolescence results in an increased level of locomotor activity, as well as a reduced number of entries into—and time spent in—the open arms of the maze (Kumari et al., 2016; Shetty and Sadananda, 2017; Amancio-Belmont et al., 2020); a pattern which has shown itself to be reversible through the use of various types of anxiolytic drugs such as Diazepam, Alprazolam y Pentylenetetrazol (Pellow et al., 1985; Sorregotti et al., 2013; Sprowles et al., 2021).

In general, the behavioral, cognitive, and neural effects resulting from social isolation can be reversed by means of resocialization (Wright et al., 1991; Chen et al., 2016; An et al., 2017), however, this method cannot reverse the effects of social isolation imposed immediately after weaning (Einon and Morgan, 1977). Nevertheless, when social isolation is applied during adolescence or adulthood, a variety of the effects caused thereby can indeed be reversed by means of resocialization (Rivera et al., 2021).

This study takes into account the potential of the social isolation to model the neurobiology of depression and anxiety; and operationalizes an increase in the consumption of high–concentration sucrose solutions as an indicator of anhedonic-type behavior potentially associated with an elevated pleasure threshold. The objective of the present study is therefore to evaluate the effect of CSI in male Wistar rats upon their anhedonic-type behavior in the SPT and their anxiogenic profile in the EPM. Additionally, the study aims to evaluate the effect of resocialization upon sucrose consumption.

## Materials and Methods

### Subjects

The present study employed 24 male adolescent Wistar rats, each weighing between 135 and 170 g (Nistiar et al., 2012), and sourced from the vivarium of the Industrial University of Santander (UIS). The test animals were given ad libitum access to food and water, and were handled only for the provision of food and daily hygiene under a controlled lighting regimen of 12 h light and darkness (with the lit period beginning at 07:00) and maintained at an ambient temperature of 22°C. The test subjects were housed either individually (in the case of the experimental group), or communally (the control group). In the former group (*n* = 12), each rat was housed in an acrylic box which allowed for visual, olfactory, and auditory contact with the other animals, but without providing an opportunity for physical contact. The remaining rats (*n* = 12) were housed in groups of six per box; these being considered as the control group. All of the experiments were performed in accordance with the approved ethical standards for animal experimentation (Congreso Nacional de la República de Colombia, 1989).

### Materials

Acrylic boxes measuring 23 × 23 × 34 cm were provided for individual housing, with stainless steel boxes of 40 × 33 × 16 cm used for communal housing. SPT: the reward response of the animals was measured by evaluating their preference for appetizing sucrose solutions over pure water (Willner et al., 1987). EPM: this maze was constructed using a four-armed wooden cross raised 50 cm from the ground. Two of the arms measured 50 × 10 cm and were open (unwalled), while the other two arms measured the same, but included walls 40 cm in height, and were open-roofed. The two open and two closed arms were arranged perpendicular to each other (Pellow et al., 1985). The frequency with which the animals entered and the time they remained, in the open arms of the maze were used as operational indicators for anxiety-type behavior in this model (Pellow et al., 1985; File and Zangrossi, 1993). The percentage of instances in which the animals entered the open arms were calculated in relation to the total number of times they entered any arm. The total number of entries into both the open and closed arms, as well as the number of times the rats crossed between arms, were taken as an operational indicator of their level of locomotor activity (Pellow et al., 1985; Botelho et al., 2007). Ethogram: a Microsoft Excel file was created to record the frequency of entries and time of stay in each of the arms of the maze (Conde, unpublished data; Ethogram is a file programmed in Excel by one of the researchers of the research group for recording behavior of rats in the Elevated Cross Maze).

### Procedure

**SCHEME 1:**
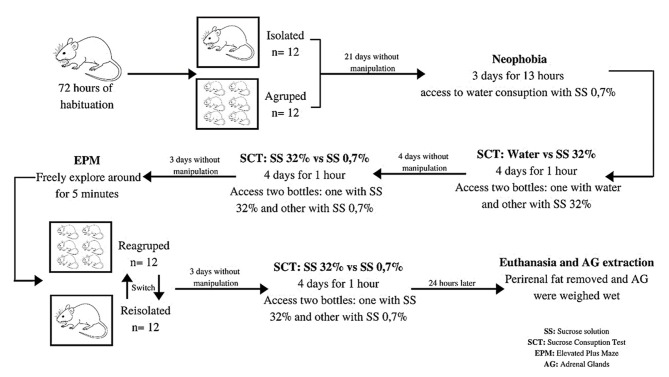
Experiment model.

Upon their arrival in the laboratory, the rats were given 72 h to adapt to their environment, after which they were randomly assigned into two housing regimens—individual and communal—for 21 days (Brenes and Fornaguera, 2009; Weintraub et al., 2010). Thereafter, they were given access to a drinking spout which provided a 0.7% SS. This spout was made available for 13 h each day (18:00–07:00) over a period of 3 days for the purpose of habituating the animals to the consumption of sucrose and avoiding possible neophobia (Hall et al., 1997). Upon the completion of these 3 days of habituation, the animals were evaluated with respect to their sucrose consumption preferences over the following four consecutive days. On each of those days, the rats were moved to the testing area and kept for 1 h in an acrylic box (with the same characteristics as those used for individual housing) where they were given access to two drinking spouts: one which provided pure water and the other a 32% sucrose solution. The consumption of the rats was measured for six animals simultaneously; three of the individually housed rats, and three of those housed communally. The schedule for these consumption evaluations was assigned randomly (Scheme 1).

Following the evaluation of their consumption preferences for the 32% sucrose solution, the animals were allowed to rest for 4 consecutive days in the same housing conditions as they had previously experienced (i.e., individual or communal), and provided only pure water to drink. Thereafter, they were once again evaluated for their preference for SS at 32% and 0.7% for a further four consecutive days, using the same evaluation procedure as described previously (Scheme 1).

Seventy-two hours after the completion of this latest round of SPT evaluation, the animals were tested *via* the EPM procedure. This test consisted of placing the animal in the center of the maze, facing towards one of the closed arms, and allowing it to explore freely for 5 min (Scheme 1). During this time, its number of entries into the open and closed arms, and the duration of its stay therein, was recorded. One entry was defined to occur when the animal positioned itself with all four of its paws within the arm of the maze (Pellow et al., 1985). Each of these sessions was monitored and recorded *via* closed-circuit television. The sessions were recorded “online” by a video camera positioned over the maze, with the videos analyzed “offline” by the same researcher, with the results recorded using the ethogram (Conde, unpublished data).

Following the completion of the latter evaluation, the animals were resocialized, with the housing regimen reversed. The animals previously housed individually were now housed communally, and those previously housed communally were placed into individual boxes. Seventy-two hours after this resocialization (Páez-Ardila and Botelho, 2014), the rats were exposed to one final round of SPT (32% and 0.7% SS) using the same protocol as previously described (Scheme 1). It was considered that the effect of re-exposing the rats to the EPM could generate a degree of acquisition and retention, with potential changes to the animals’ behavior due to their experience in the first exposure (Rodgers et al., 1996; Lamprea et al., 2000; Belviranli et al., 2012). This, in turn, could result in an intensification of fear and generate a state of phobia (File and Zangrossi, 1993), thus, the effect of the resocialization upon the rats’ anxiogenic profile was not evaluated. Finally, 24 h after the last day of evaluation using the SPT (at 32% vs. 0.7%), the rats were euthanized, and their adrenal glands (AG) were extracted. The periadrenal fat was removed, and the wet weights of the glands were measured as a physiological indicator of stress (Selye, 1936; Grippo et al., 2003; Rygula et al., 2005; Scheme 1).

## Analysis of The Results

To evaluate the effects of CSI upon sucrose consumption for days 1 (D1), 2 (D2), 3 (D3), and 4 (D4) of the first and second exposures, a 3-way analysis of variance (ANOVA) was carried out, followed by a *t*-test for multiple comparisons (using the Holm–Sidak method).

Factor 1 refers to the GROUP (type of housing: individual or communal); factor 2, to the CONCENTRATION of the sucrose solutions (pure water, 0.7%, and 32%); and factor 3, to the DAY of exposure. To evaluate the effects of CSI on the anxiety-type behavior in the EPM and the weight of the AG, analysis was performed using *t*-tests for independent samples; parametric or non-parametric, depending on the distribution of the data and the homogeneity of the sample (student’s *t*-test or Mann–Whitney, respectively). These tests compared the two groups: isolated (AISL) and communal (AGRUP).

Finally, to evaluate the effects of the 72 h of resocialization upon the consumption of sucrose for days 1, 2, 3, and 4; a three-way ANOVA test was used, followed by a *t*-test for multiple comparisons (Holm–Sidak method). Factor 1 refers to the GROUP [type of housing: REAIS (Animals initially communal and later isolated) vs. REGRUP (Animals initially isolated and later communal)]; factor 2, to the CONCENTRATION of the sucrose solutions (0.7% and 32%); and finally, factor 3 to the DAY of exposure. In all statistical analyses, significance was defined as *p* < 0.05. All of the statistical analyses were performed using SigmaStat software (Systar Software Inc., 2016).

## Results

### Consumption of Pure Water vs. 32% Sucrose Solution

For the first exposure (pure water vs. 32% sucrose solution), it was found that the factors of group (*F*_(1,176)_ = 29.667, *p* < 0.001), concentration (*F*_(1,176)_ = 494.048, *p* < 0.001), and day (*F*_(3,176)_ = 9.583, *p* < 0.001), as well as the interaction between group and concentration (*F*_(1,176)_ = 56.397, *p* < 0.001) and the interaction between concentration and day (*F*_(3,176)_ = 19.412, *p* < 0.001) were sources of variation. The Holm–Sidak *t*-test for multiple comparisons showed that the two groups, both isolated (*t* = 21.027) as well as communal (*t* = 10.407), consumed more of the 32% sucrose solution than pure water. Nevertheless, the isolated rats consumed more of the sucrose solution (*t* = 9.162) than did the other group.

With respect to the per day consumption of SS, the analyses demonstrated that the isolated animals consumed more on D4 as compared to D1 (*t* = 4.873; *p* = 0.009) and D2 (*t* = 2.896; *p* = 0.01), however, no statistically significant differences were found in the consumption of SS between D4 and D3 (*t* = 2.193; *p* = 0.017). Similarly, they consumed more on D3 as compared to D1 (*t* = 2.68; *p* = 0.013). For their own part, the communally housed animals consumed more SS on D3 than on D1 (*t* = 3.077; *p* = 0.009). Also, specifically on D2 (*t* = 3.138; *p* = 0.05) and D4 (*t* = 4.691; *p* = 0.05), it was the isolated animals who consumed more.

**Figure 1:**
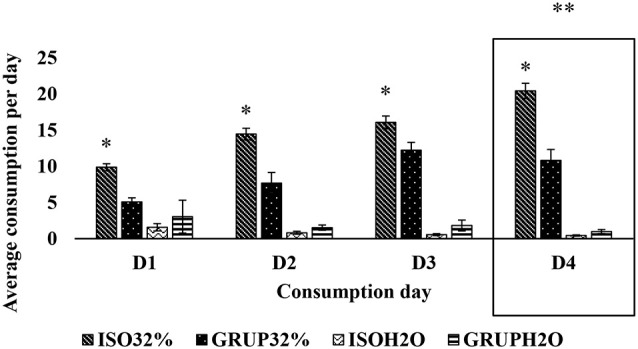
Consumption of pure water and 32% sucrose solution by groups on D1, D2, D3, and D4. Average consumption (PROM ± EE) of pure water vs. 32% SS on D1, D2, D3, and D4, of the groups ISO32% (isolated 32%); GRUP32% (grouped 32%); ISOH2O (isolated water); and GRUPH2O (grouped water). *Higher consumption of 32% SS tan water in isolated D1, D2, D3 y D4. **Higher consumption of 32% SS in isolated and grouped on D4 than D1, D2, and D3 (Holm–Sidak Test *t, p* < 0.05).

In accordance with the previous results, it can be seen that both the communally housed and isolated animals consumed more of the SS on D4 (*t* = 8.446; *p* = 0.009), D3 (*t* = 6.923; *p* = 0.01), and D2 (*t* = 3.725; *p* = 0.017), as compared with D1. It is also evident that on D1 (*t* = 5.339; *p* = 0.05), D2 (*t* = 10.242; *p* = 0.05), D3 (*t* = 13.427; *p* = 0.05), and D4 (*t* = 15.447; *p* = 0.05), the animals consumed more of the 32% SS than of pure water (Figure 1).

### Consumption of 32% vs. 0.7% Sucrose Solution

The 3-way ANOVA carried out for the analysis of the second exposure (consumption of 0.7% vs. 32% sucrose solution), revealed statistically significant differences related to the group (*F*_(1,176)_ = 64.413, *p* < 0.001) and concentration (*F*_(1,176)_ = 1051.101, *p* < 0.001) variables. It also indicated that the interaction between these two factors (*F*_(1,176)_ = 52.138 *p* < 0.001) was a source of variation.

According to the Holm–Sidak *t*-test, both groups, the isolated (*t* = 28.031; *p* < 0.05) as well as the communally housed rats (*t* = 17.819; *p* < 0.05), consumed more of the 32% SS than the 0.7% solution. Nevertheless, the group of isolated rats exhibited a higher consumption of the 32% solution (*t* = 10.781; *p* < 0.05) than that of the communally housed animals.

**Figure 2:**
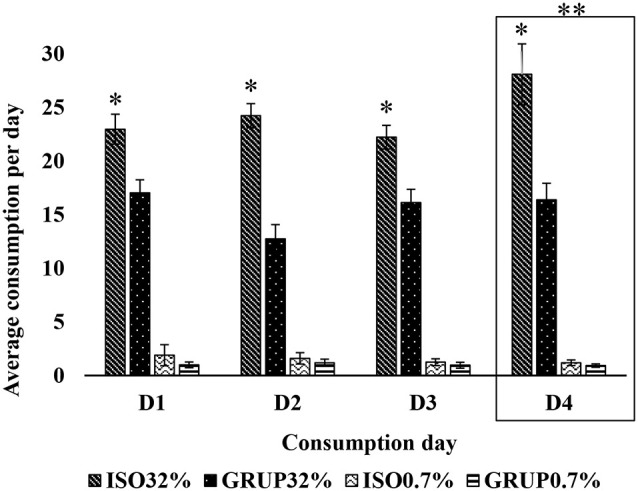
Consumption of 32% sucrose solution vs. 0.7% sucrose solution by groups on D1, D2, D3, and D4. Average consumption (PROM ± EE) of 32% SS vs. 0.7% SS on D1, D2, D3 and D4, in the groups ISO32% (isolated 32%); GRUP32% (grouped 32%); ISO0.7% (isolated 0.7%); GRUP0.7% (grouped 0.7%). *Higher consumption of SS in ISO32% than ISO0 7%. **Higher consumption of 32% SS in isolated and grouped on D4 than D1, D2 and D3 (Holm–Sidak Test *t, p* < 0.05).

Similarly, socially isolated animals consumed more than the grouped animals on days D1 (*t* = 2.957; *p* = 0.05), D2 (*t* = 5.152; *p* = 0.05), D3 (*t* = 2.762; *p* = 0.05), and D4 (*t* = 5.181; *p* = 0.05). On D4, a higher consumption of SS 32% was recorded as compared to D2 (*t* = 3.246; *p* = 0.009) and D3 (*t* = 2.643; *p* = 0.01); and on days D1 (*t* = 16.07; *p* < 0.05), D2 (*t* = 14.791; *p* < 0.05), D3 (*t* = 15.651; *p* < 0.05), and D4 (*t* = 18.33; *p* < 0.05), the animals of both groups consumed a greater quantity of the 32% sucrose solution (Figure 2) than of the 0.7% solution.

Taken together, the results obtained from the SS consumption tests demonstrate that both in conditions of CSI or communal housing, the subject animals prefer to consume more of the sweeter solution (32%) than of the less sweet (0.7% or water); although in the socially isolated rats, this preference was seen to be more pronounced than in the communally housed ones.

### Exploration in the Elevated-Plus-Maze (EPM)

**Figure 3:**
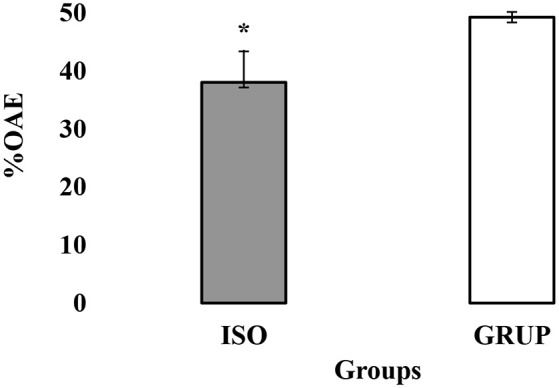
Exploration of the open arms of the elevated-plus-maze (EPM). Exploration of the open arms of the EPM. OAE y %OAE (PROM ± EE) in EPM, in the groups ISO (isolated) y GRUP (grouped). *Less %OAE in ISO than GRUP; Mann–Whitney Test *t, p* < 0.05.

In general terms, no statistically significant differences were found between the time and percentage of stay in the open arms (TBA) and closed arms (TBC) of the EPM between the rats assigned to the AISL and AGRUP groups (*p* > 0.05). Nonetheless, the student’s *t*-test indicated that the animals from the AISL group entered the open arms with lower frequency (EBA; *t* = −2.314, *p* = 0.030) and %EBA (*U* = 110.500, *p* = 0.0024) than those assigned to AGRUP (Figure 3). Logically, then, the AGRUP rats entered the closed arms of the maze (%EBC) less often than those of the AISL group (*t* = −2.231, *p* = 0.036).

The student’s *t*-test revealed a statistically significant difference between the AISL and AGRUP groups with respect to locomotor activity. The AISL animals exhibited a lower number of total entries (EBA+EBC; *t* = −2.283, *p* = 0.032) and number of crossings of the EPM (*t* = −2.378, *p* = 0.026) than the AGRUP animals.

### Consumption of 32% vs. 0.7% Sucrose Solution After 72 h of Resocialization

To analyze the second exposure to 32% vs. 0.7% SS after 72 h of resocialization, a 3-way ANOVA was carried out, which indicated that the factors of group (*F*_(1,176)_ = 13.815, *p* < 0.001), concentration (*F*_(1,176)_ = 2490.816, *p* < 0.001), and day (*F*_(3,176)_ = 22.563, *p* < 0.001) were all sources of variation. Statistically significant differences were also found for the interactions between group and concentration (*F*_(1,176)_ = 13.623, *p* < 0.001), group and day (*F*_(3,176)_ = 2.954, *p* = 0.034), and concentration and day (*F*_(3,176)_ = 25.654, *p* < 0.001).

The Holm–Sidak *t*-test indicated that the two groups, REAIS (*t* = 32.68; *p* < 0.05) and REGRUP (*t* = 37.9; *p* < 0.05), consumed more of the 32% solution than of the 0.7%. Nevertheless, it was the REGRUP group that consumed a greater quantity of the 32% solution (*t* = 5.238; *p* < 0.05). Thus, the animals originally subjected to CSI for 21 days consumed a greater quantity of the sweeter solution, suggesting that their subsequent resocialization did not reverse the effect of chronic stress upon their preference for 32% SS.

**Figure 4:**
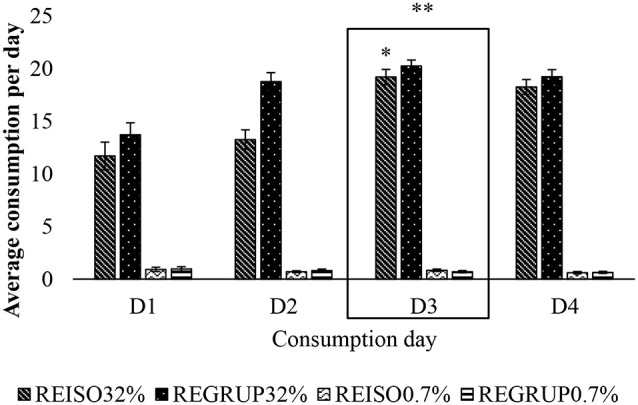
Average consumption of 32% sucrose solution vs. 0.7% sucrose solution on D1, D2, D3, and D4 after exposure to 72 h of resocialization. Average consumption (PROM ± EE) of 32% SS vs. 0.7% SS on D1, D2, D3, and D4, in the groups REISO32% (Grouped animals that became isolated 32%); REGRUP32% (Isolated animals that became grouped 32%); REISO0.7% (Grouped animals that became isolated 0.7%); and REGRUP0.7% (Isolated animals that became grouped 0.7%) after 72 h of resocialization. *Higher consumption of SS in ISO32% than ISO0.7% on D4, D2, D1 and D3. **Higher consumption of SS in ISO32% than ISO0.7% on D3 than D4, D2 and D1 (Holm–Sidak Test *t, p* < 0.05).

The animals were found to consume more of the sucrose solution on days D3 (*t* = 10.943; *p* = 0.009), D4 (*t* = 9.406; *p* = 0.01), and D2 (*t* = 3.304; *p* = 0.017), as compared to D1; and more on D3 (*t* = 5.799; *p* = 0.013) and D4 (*t* = 4.262; *p* = 0.025) than on D2. As well, it can be seen that on days D1 (*t* = 18.325; *p* < 0.05), D2 (*t* = 23.748; *p* < 0.05), D3 (*t* = 29.527; *p* < 0.05), and D4 (*t* = 28.217; *p* < 0.05) the animals consumed more of the 32% sucrose solution than the 0.7%, most notably so on D3 (Figure 4).

**Figure 5:**
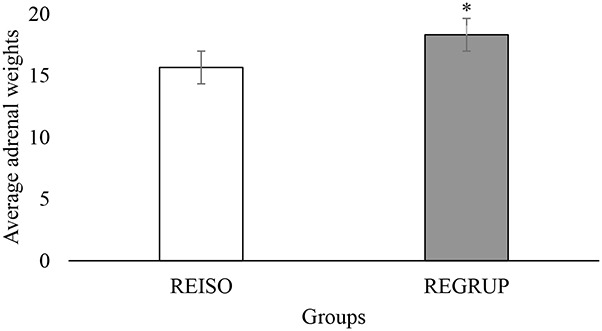
Average weight of adrenal glands (AG) by group. Average weight (PROM ± EE) of the AG in the REISO (grouped animals that became isolated) and REGRUP (isolated animals that became grouped) groups. *Greater weight of the AG in REGRUP than REISO (Student Test *t, p* < 0.05).

Considering the importance of having objective measurements of emotion in rats, the present study used the weight of the AG (measured as a function of the animal’s total weight 24 h after the final SPT) as a physiological indicator of its response to stress. Per the student’s *t*-test, the animals from the REGRUP group exhibited heavier glands (*t* = −2.651, *p* = 0.002) than those of the animals from the REAIS group (Figure 5). This is to say that the animals which were initially isolated for 21 days exhibited heavier glands than those of the animals which were originally housed communally. Once again, and in alignment with the results from the SPT consumption tests, it appears that resocialization did not reverse the effect of CSI upon the rats’ physiological response to stress.

## Discussion

The idea of the current study is to contribute to the knowledge about the consequences of CSI in male Wistar rats on their anhedonic-type behavior in the SPT and their anxiogenic profile in the EPM. Moreover, the study looks forward to evaluating the effect of resocialization upon sucrose consumption. In general terms, it was seen that both the communally housed and isolated animals demonstrated a preference for the consumption of 32% sucrose solution, however, the animals subjected to CSI consumed more of the sweeter solution and exhibited heavier AG than the animals from the AGRUP control group. Furthermore, they demonstrated an anxiogenic profile in the EPM test. Resocialization reversed neither the increased preference for 32% SS nor the weight of the AG.

These results align well with other studies which show that exposure to chronic stressors (Gawali et al., 2017; Nelemans et al., 2017; Steudte-Schmiedgen et al., 2017), especially social deprivation, leads to a series of behavioral changes, including some related to the reward system (Pritchard et al., 2013). The results of the present study are thus in agreement with those of Brenes Sáenz et al. (2006) and Brenes and Fornaguera (2008) who showed that rats subjected to CSI immediately after weaning consume more 32% sucrose solution than pure water. These authors affirm that this increased consumption of 32% SS was found to be associated with sensitivity to reinforcement, and can be understood as a sign of behavioral desperation (Brenes Sáenz et al., 2006; Brenes and Fornaguera, 2008). In support of the above, Hall et al. (1997) utilized CSI as a stressor followed by evaluation of varied concentrations of sucrose solutions (0.7%, 2.1%, 7%, 21%, and 34%). These authors used two schemes to present the test subjects with the solutions; the first in ascending order of concentration, and the latter in a descending order. It was discovered that rats subjected to CSI consumed more 34% SS in both schemes. These results were discussed in light of the effects of CSI upon the animals’ reactiveness to highly appetizing concentrations of SS and emphasized the involvement of the dopaminergic system in the nucleus accumbens as a modulator of the incentivizing effects of sucrose (Wright et al., 2020).

Furthermore, in a study by Cortés et al. (2005), it was shown that female rats subjected to CSI consumed more 32% SS than 0.7% solution or pure water; as well as finding that animals housed individually exhibited a heightened preference for 32% SS as compared to animals housed communally. The authors demonstrated that CSI resulted in depression and anxiety-type behaviors, which appeared in both the SPT and EPM tests, respectively (Cortés et al., 2005).

Notably, the consumption of 32% SS in the present study was incremented in an ascending fashion over the full course of the experiment; that is, at both the first and second exposures, with consumption on D4 being higher than consumption during the preceding days. This result aligns with those of related studies, which found the consumption of sweet (32%) sucrose solutions to follow a gradually increasing path (Hall et al., 1997; Sammut et al., 2002; Cortés et al., 2005) while that of water or less sweet SS to diminish over the course of several days (Hall et al., 1997; Ayuso-Mateos et al., 2012). Martínez et al. (2008) hypothesize that the preference for sweeter solutions could demonstrate the importance of pleasure thresholds along the course of “anhedonic-type behavior.”

Páez-Ardila and Botelho (2014) evaluated the effects of CSI upon SS consumption in young orchiectomized adult rats and found that independently of treatment (i.e., castrated or uncastrated), those animals which were subjected to CSI exhibited a greater consumption of 32% SS than those housed communally, although uncastrated rats exhibited the highest overall consumption. These results demonstrate the importance of the hormonal factor upon the course of Major Depressive Disorder (MDD), although various human studies on the subject have found that reduced levels of testosterone appear to be associated with the appearance of this disorder (Ayuso-Mateos et al., 2012; Rice and Sher, 2017). According to Rice and Sher (Rice and Sher, 2017), the tendency towards MDD associated with a hormonal component could be related to the age of the subjects, since young rats with the highest testosterone levels have been found to be most closely associated with “depressive-type behavior” (Torres-González et al., 2009). Furthermore, in a study carried out by Rodríguez et al. (2012), male rats were subjected to a CSI model, with those animals thus treated tending to consume a higher quantity of 32% SS. Additionally, the consumption of sweet SS was greater in animals subjected to the forced-swim test before the SPT, a finding which highlights the stressor effect of the forced-swim model.

Contrary to the results of the present study, Papp et al. (2016) found that chronic stress led to reduced ingestion of 1% sucrose solution. This behavior was normalized by means of treatment with imipramine, ketamine, rivastigmine, and donepezil; which was interpreted to indicate their antidepressant effects. Similarly, Qin et al. (2017) and Sun et al. (2017) stated that treatment with melatonin was able to reverse this reduced consumption of low–concentration SS, as a consequence of deficiencies in reward systems produced by chronic unpredictable mild stress (CUMS), and which have traditionally been associated with “anhedonic-type behavior” (Willner et al., 1987; Qin et al., 2017; Sun et al., 2017; He et al., 2020). Also, Muscat et al. (1991) demonstrated that Raclopride exhibits an effect upon the behavior of male rats, creating anhedonic-type behavior, as shown by their preference for the consumption of high-concentration (34%) SS over 0.7% and 7% concentrations. These results indicate that the mesolimbic dopaminergic system plays a fundamental role in the response to reward stimuli since Raclopride is a dopamine receptor antagonist. Similarly, Wang et al. (2020) reported that CUMS also led to a reduction in body mass and depression-type behavior, as evidenced by a reduction in the consumption of 1% sucrose solution; a pattern reversible using fluoxetine.

Various methodological factors could explain the reduction of low–concentration SS consumption in the studies mentioned above; among them, the type of stressor utilized, the consumption measurement scheme, and the age of the test subjects. Additionally, the effect could occur due to the withholding of water and food before exposure to the SS, as this could affect the animals’ ingestion.

Furthermore, it is noteworthy that CSI which occurs in young animals or immediately after weaning exhibits a distinct form of impact upon anhedonic-type behavior, which could, in turn, result in elevated pleasure thresholds (Martínez et al., 2008; Díaz et al., 2010; Páez-Ardila and Botelho, 2014). This observation finds basis in studies which show that the resocialization of animals subjected to SCI immediately after weaning, as adolescents, or as young animals is not capable of reversing the emotional behavior produced by this model, and results in behavioral deficits in the long term (Einon and Morgan, 1977; Whitaker et al., 2003).

From a pharmacological point of view, various studies have indicated that chronic treatment with antidepressants only reverses the pattern of consumption of 32% sucrose solution (Sammut et al., 2002). Some, such as Brenes and Fornaguera (2009), demonstrate that the increase in consumption of sweet (32%) SS produced by CSI can be reversed by fluoxetine; a finding which is consistent with the involvement of the serotonergic system in the regulation of motivated behavior towards a palatable incentive.

Taking into account the incongruencies found in the operational definition of anhedonia, it is possible that these can be attributed to the stress model used to induce anhedonic-type behavior (Brenes Sáenz et al., 2006), as the majority of studies that have linked anhedonic-type behavior to reduced SS consumption have utilized the CUMS model, while those that have linked it to increased consumption have used the CSI, as in the present study.

Another possible cause may arise from the sex of the animals, due to the differential susceptibility to stress responses caused thereby (Liang et al., 2008; Burke et al., 2016). Page et al. (2016) reported that among the female and male rats exposed to a model of social defeat, the female animals exhibited anhedonic-type behavior, this being taken as a reduction in their consumption of low–concentration SS. In an opposite finding, however, Burke et al. (2016) observed that when animals of both sexes were subjected to stress inducers, the males exhibited a reduction in their consumption of a low–concentration SS, which was taken as an operational definition of anhedonic-type behavior.

Additionally, Liang et al. (2008) found that, upon subjecting male and female rats to a chronic mild stress model, females in heat consumed a greater amount of SS compared to the males and the females not in heat. This result suggests that the females exhibited increased susceptibility to stressors relative to their stage of the estrous cycle (Liang et al., 2008). Supporting these results, Cortés et al. (2005) showed that females subjected to CSI exhibited an increased consumption of high–concentration sucrose solutions. These findings could be explained by the action of gonadal hormones, which play a fundamental role in regulating mood (Martínez-Mota et al., 2012).

In the same manner, in studies carried out with human test subjects, even though women exhibit higher indices of MDD prevalence than men, they also demonstrate a more effective recovery by means of antidepressants and other treatments; while men respond more slowly to therapy (Liang et al., 2008; Díaz Sotelo, 2016) and exhibit a higher incidence of suicidal ideation and behavior; especially when this occurs simultaneously with low testosterone levels experienced by the senior population (Díaz Sotelo, 2016; Nelemans et al., 2017), as well as the heightened levels found in adolescents and young adults (Rice and Sher, 2017).

In line with the marked comorbidity which exists between depression and anxiety (Nelemans et al., 2017; Sangenstedt et al., 2017; Steudte-Schmiedgen et al., 2017), this study sought to evaluate the effect of CSI upon the animals’ exploration of the EPM. Taken together, the results of the present study align with those of previous studies which show CSI to produce an anxiogenic profile on the EPM (Pellow et al., 1985; Papp et al., 2016; Bavley et al., 2017; Biala et al., 2017; Sun et al., 2017; Viana Borges et al., 2019). According to some authors, male adolescent rats exhibited less exploration of the open arms and lower levels of locomotor activity on the EPM, thus demonstrating a reaction to stress. These authors suggest that this anxiogenic profile may be modulated by hormonal states (Liang et al., 2008; Viana Borges et al., 2019). This proposal is supported by similar results in female rats by Kumari et al. (2016), who suggested that in light of the differences attributable to sex, although greater presence of anxious behaviors are found in females, it is, in fact, males who, when subjected to chronic stressors such as CSI, increase and maintain high levels of these behaviors. Weintraub et al. (2010) add that females exhibited higher percentages of entries into the open arms of the EPM (vs. the closed arms) when compared to males; indicating that despite being subjected to the same stressor, the males demonstrated higher anxiety levels than the females.

Finally, the present study sought to determine the effect of resocialization upon SS consumption preferences. The results showed that the anhedonic-type behavior of the male rats produced *via* CSI was not reversed by 72 h of resocialization. These results align with the literature, which indicates that resocialization is not able to reverse the adverse effects caused by CSI such as sensorimotor deficits, difficulty with the fear response, and circadian rhythm irregularities (Einon and Morgan, 1977; Whitaker et al., 2003; Weintraub et al., 2010). Specifically, resocialization did not reverse aggressivity and acute anxiety produced by CSI applied immediately following weaning in male rats (Walker et al., 2008). In a similar fashion, it has been demonstrated that the behavioral effects of acute social deprivation can be reversed by resocialization, but not those produced by chronic exposure to the same (Einon and Morgan, 1977).

On the other hand, it has been demonstrated that resocialization can reverse some of the effects produced by CSI (Tulogdi et al., 2014). For example, in a study conducted by Tulogdi et al. (2014), despite the fact that resocialization did not reverse the aggressivity produced by CSI applied immediately after weaning, adult animals were able to overcome the resulting deficiencies in pro-social behavior. This discrepancy among the results in the aforementioned studies may be explained by methodological differences, which include the sex and exact breed of animal, the duration of social isolation and the age at which it was applied, the physical conditions of housing, as well as the number of rats that are housed communally (Einon and Morgan, 1977; Hellemans et al., 2004).

Additionally, pioneering researchers in this field have suggested that resocialization is only effective when it is implemented for twice as long as the preceding isolation; this is to say, in the present study, the animals should have been resocialized for approximately 80 days, considering that they underwent an experimental period of 40 days between their initial isolation and the end of the protocol. Nevertheless, in a study conducted by Martínez et al. (2008), it was reported that resocialization for a period of 72 h did successfully reverse the effects of CSI upon anhedonic-type behavior but not of anxiety, as the latter may respond to the particularities of an array of disorders. As well, many studies report that the effects of CSI applied after weaning or during adolescence are not reversible *via* resocialization (Selye, 1936; Einon and Morgan, 1977), in contrast to when the animal is subjected to CSI in adulthood (Hellemans et al., 2004).

Finally, the animals that were originally isolated for 21 days exhibited heavier AG than those that were initially housed together. This is to say; 72 h of resocialization did not reverse the effect of CSI upon their physiological stress response. In a study conducted by Rygula et al. (2005), it was found that rats exposed to a model of social defeat produced AG heavier than those of the control group. Similarly, Grippo et al. (2003) demonstrated that rats subjected to mild chronic stress over the course of 4 weeks exhibited heavier AG than those of the control group. These results align with various studies which demonstrate that different stressors lead to heavier rodent AG as a consequence of the activation of the hypothalamic-pituitary-adrenal (HPA) axis (Selye, 1936; Rygula et al., 2005; Pariante and Lightman, 2008; Walker et al., 2008; Díaz et al., 2010; Gawali et al., 2017). These findings highlight the importance of stress in the etiology of anxiety and depression (Pariante and Lightman, 2008; Gawali et al., 2017; Sangenstedt et al., 2017; Steudte-Schmiedgen et al., 2017).

Overall, the results of the present study reinforce the hypothesis that anhedonic-type behavior can be associated with an elevated pleasure threshold. Future studies may explore this hypothesis from a neurochemical point of view, using the same experimental protocol. Additionally, it would be desirable to study the difference which arises with respect to the effect of CSI upon anhedonia and anxiety profiles as relates to sex, and evaluate whether resocialization, for a period of time longer than that of isolation, is able to reverse the behavioral and physiological effects produced by social deprivation.

## Conclusion

In the present study, it was demonstrated that exposure to CSI produces changes in the natural reward sense, as exhibited by a greater consumption of high–concentration sucrose solutions (which was not reversible through resocialization) and an anxiogenic profile in male adolescent rats.

## Data Availability Statement

The datasets presented in this study can be found in online repositories. The names of the repository/repositories and accession number(s) can be found below: https://repository.upb.edu.co/handle/20.500.11912/7769.

## Ethics Statement

The animal study was reviewed and approved by Ethics Committee of Universidad Pontificia Bolivariana.

## Author Contributions

All authors contributed to the article and approved the submitted version.

## Conflict of Interest

The authors declare that the research was conducted in the absence of any commercial or financial relationships that could be construed as a potential conflict of interest.
